# Comparative Analysis of Dentoskeletal Changes of the Twin Block Appliance and the AdvanSync2 Appliance in Treatment of Skeletal Class-II Malocclusion in Pakistani Population: A Randomized Clinical Trial

**DOI:** 10.1055/s-0041-1739543

**Published:** 2021-12-15

**Authors:** Fareena Ghaffar, Abdullah Jan, Obaid Akhtar, Alaina T. Mughal, Rooma Shahid, Hafiza Z. Shafique, Khadija Bibi, Sundas Mehmood, Nimra Afgan, Rumeesha Zaheer

**Affiliations:** 1 Department of Orthodontics, Armed Forces Institute of Dentistry, Rawalpindi, Pakistan; 2 Department of Prosthodontics, HBS Dental College, Islamabad, Pakistan

**Keywords:** AdvanSync2 appliance, dentoskeletal changes, fixed functional appliances, removable functional appliances, skeletal class-II malocclusion, twin block appliance

## Abstract

**Objective** This study aimed to compare dentoskeletal changes in skeletal class-II malocclusion with removable twin block appliance and fixed AdvanSync2 appliance.

**Materials and Methods** A prospective randomized clinical trial was conducted over a span of 1 year at AFID at Rawalpindi. Thirty patients with skeletal class-II malocclusion, 16 males (53.3%) and 14 females (46.6%), were randomly selected and divided in two equal groups (15 each) to be treated with either fixed functional appliances (FFAs) or with removable functional appliances (RFAs). Out of 30 patients, 15 between cervical vertebral maturation (CVM) stages of 2 and 3 were treated with RFA (twin block appliances) and remaining 15 between CVM stages of 4 and 5 were treated with FFA (AdvanSync2 appliances). Pretreatment (T _1_ ) and posttreatment (T _2_ ), angular variable, and linear variable were measured to compare the dentoskeletal effects between the two groups.

**Statitical Analysis** Paired sample t-test was used to assess significant difference between variables at T1 (Pre-treatment) and T2 (Post-treatment) stage for both RFA and FFA group. Comparison among the RFA and FFA group was made using non-parametric Mann-Whitney U Test. IBM SPSS version 25.0 was used for evaluation.

**Results** No significant difference was found in angular variables between the RFA and FFA groups ( *p* > 0.05) with the exception of linear variables. Sella-posterior nasal spine (S-PNS) length significantly increased and Jarabak's ratio significantly decreased for FFA group ( *p* = 0.010 and 0.045, respectively), when compared with RFA group.

**Conclusion** Both the appliances, twin block (RFA) and AdvanSync2 (FFA), are effective for correction of skeletal class-II malocclusion. Both the appliances produced similar effects in the sagittal plane but for better vertical control twin block should be the appliance of choice. AdvanSync2 appliance could be preferred over twin block appliance when dentoalveolar and slight retrusive effect on the maxilla is desired especially for individuals in postpubertal growth spurt.

## Introduction

The most frequently reported cases in orthodontics are of class-II malocclusion. 1 According to Tariq et al, 2 41% of total orthodontic cases in Pakistani population are of class-II malocclusion. Class-II malocclusion occurs as a result of maxillary protrusion, or mandibular retrusion or combination of both which can be corrected by treating the skeletal and dentoalveolar discrepancies. Out of many recommended treatment options, one can make use of either removable and/or fixed functional appliances (FFAs). 3 Headgear is one of the classic appliances used for the correction of class-II malocclusion. 4 Other removable functional appliances (RFAs) include Frankel's functional regulators (FR), Balter's bionator, and Sander's bite jumping appliances. Jasper jumper, Herbst appliance, and mandibular anterior repositioning appliance (MARA) are some of the FFAs used in treatment of class-II discrepancy. All these modalities are designed to modify the arches by reorienting their position in both sagittal and vertical dimensions to bring about correction of main features of class-II malocclusion. 5 6

AdvanSync2 (Ormco Co., Glendora, California, United States), an FFA developed by Terry Dischinger in 2008, is a recent modification of Herbst appliance. It is a molar-to-molar appliance that connects maxillary and mandibular arches by telescopic rods. It is less bulky than the conventional Herbst appliance and has shown reduction in treatment duration up to 6 to 9 months. It is much more acceptable by the patients as they complain less about sores and discomfort and is esthetically pleasant since it is not visible in the mouth. 7 The appliance helps to advance the mandible in a constant forward position to stimulate remaining growth in a more favorable direction. 8

Twin block appliance (RFA) was introduced by William Clark in 1988. Many modifications for this appliance have been introduced lately. It has been named twin block for the characteristic of two unattached maxillary and mandibular plates with acrylic bite blocks which make a 70-degree angle when in contact with each other. The retention of the appliance is via Adam's clasps, and it plays a vital role in the treatment of mandibular retrognathia. 9 This treatment modality can be performed both at an early, as well as delayed, age for correction of class-II malocclusion. However, delayed treatment was found to be much better for the patients in terms of less orthodontic visits. 10

Hence, the objective of this study was to compare dentoskeletal changes in skeletal class-II malocclusion induced due to treatment with removable twin block appliance and fixed AdvanSync2 appliance.

AdvanSync2 is a new treatment modality in orthodontics, and no relevant literature could be found with regard to this appliance in Pakistani population as yet. Fig. S1 (available in online version only) and Table S1 (available in online version only) show Consolidated Standards of Reporting Trials (CONSORT) to facilitate transparent reporting of clinical trial.

## Materials and Methods

Permission was taken from the ethical review committee of the AFID at Rawalpindi (reference number: 905/Trg-ABP1k2) prior to the conduct of study. A prospective randomized clinical trial was performed in 2 years' duration, from March 2019 to March 2021, at the Department of Orthodontics, AFID, Rawalpindi.

Thirty patients with skeletal class-II malocclusion, 16 males (53.3%) and 14 females (46.6%), were randomly allocated in a 1:1 ratio into two equal groups (15 in each group) to be treated with either AdvanSync2 FFA or with twin block RFAs. Out of 30 patients, 15 between cervical vertebral maturation (CVM) stages of 2 and 3 were treated with RFA (twin block appliances) and remaining 15 were between CVM stages of 4 and 5 with FFA (AdvanSync2 appliances).

Sample size was 15 patients for each group, calculated by using power analysis software when the power of study was 80%, level of significance 0.05, and the detected difference kept at 0.8. The inclusion criteria for patients were class-II division-1 malocclusion with mandible being placed backward (sella–nasion point B [SNB] angle <78 degrees), convex facial profile, A point, nasion, B point (ANB) angle to be >4 degrees, overall good oral health, no previous orthodontic treatment being done, and peak of pubertal growth at the start of treatment. Patients presenting with any developmental defects, asymmetrical facial profile, and impacted/missing/supernumerary or transposed teeth were excluded from the study.

For the fabrication of twin block appliances (RFA), alginate impressions were taken and casts were poured over which bite blocks were constructed. Bite registration was done by advancing the mandible at the desired position. Self-curing acrylic blocks with inclined guiding planes on both maxillary and mandibular plates were constructed to guide anterior positioning of the mandible on closure. These acrylic blocks were subjected to sequential grinding to promote tooth eruption along with the advancement. The upper and lower bite blocks were interlocked at 70 degrees. Clasps on upper molars and premolars and lower premolars and incisors were added. A labial bow was added on the upper arch. Springs were added to move individual teeth. The patients were asked to wear appliances for 14 to 16 hours every day. All RFA were designed by the same orthodontic technician (J.M.).

For the AdvancSync2 appliance (Ormco Co., Glendora; FFA), prefabricated bands were selected from the kit of appropriate sizes for each patient. In contrast to Herbst appliances, no trans-palatal archs (TPAs) or lingual arches were given as AdvanSync2 had built in activation system. 11

Evaluation of the participants was done by 4 weeks' interval. Duration of appliance wear was from 6 to 9 months followed by a retention period of 3 months and total treatment duration was between 20 and 24 months (which includes final detailing and finishing).

**Fig. 1:**
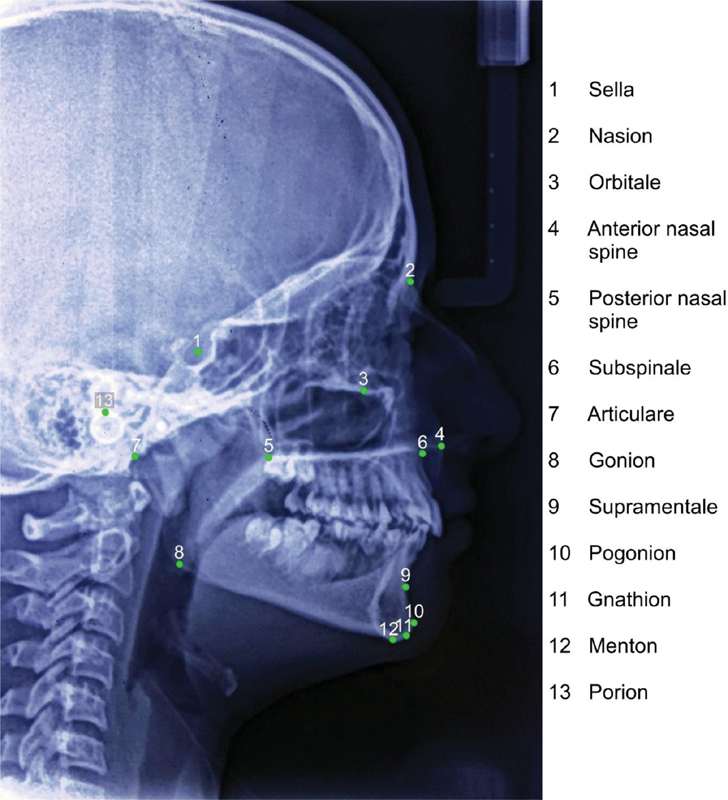
Landmarks used to assess angular and linear variable.

Data were collected at the start (T _1_ ) and by the end of treatment (T _2_ ) after functional therapy before detailing of occlusion at 9 to 12 months. Lateral cephalometric radiographs at T _1_ and T _2_ in natural head positions were obtained and drawn by conventional hand tracing method by a single author. Twenty-six angular and linear variables were then measured at these treatment intervals and dentoskeletal changes in patients of skeletal class-II malocclusion induced due to the two appliances were compared separately at T _1_ and T _2_ as defined in Table 1 . Fig. 1 shows important landmarks used to calculate these angular and linear variables.

**Table 1:** Definitions of angular and linear variables

Angular variables	Definitions
Sella–nasion point A angle (SNA)	Angle between SN to point A that specifies either the maxilla is normal, prognathic, or retrognathic
SN point B angle (SNB)	Angle between SN to point B, that specifies either the mandible is normal, prognathic, or retrognathic
ANB angle	The difference between SNA and SNB angle that tells the magnitude of discrepancy between the maxilla and mandible
SN–mandibular plane (SN–MP) angle	SN to MP angle to evaluate the vertical growth pattern using anterior cranial base as reference plane
SN–palatal plane (SN–PP) angle	Angle between SN and PP, indicating rotation of the maxilla
SN–occlusal plane (SN–OP) angle	Angle between SN and occlusal plane, indicating the relation of cranial base to occlusal plane
Saddle angle	Angle between SN to articulare (Ar), specifying the relationship between anterior and posterior cranial bases
Ar angle	Angle between upper and lower parts of posterior contours of the facial skeleton
Gonial (Go) angle	Angle formed by the junction of the posterior and lower borders of the mandible
Y-axis	Sella–gnathion (Gn) to Frankfurt horizontal plane, explains the direction of mandibular growth
Upper incisor–SN (UI–SN)	Angle formed by drawing a line between the long axis of upper incisors and SN plane
Upper incisor–PP (UI–PP)	Posteroinferior crossing angle of upper incisor axis with PP
Incisor MP angle (IMPA)	Junction of MP with a line passing through the incisal edge and the apex of the root of mandibular central incisor
Inter incisor angle (IIA)	Angle formed between the long axis of upper and lower incisors
Linear variables	
Jarabak's ratio	Ratio between anterior and posterior facial heights
SN	The distance between sella and nasion
Mandibular corpus length (MCL)	The distance measured between gonion (Go) and Gn
S–Ar	The distance measured from sella to Ar point
Ar–Go	The distance taken from Ar to Go
Go–menton (Go–Me)	The linear distance between Go and Me
N–Me	The measure of distance between N and Me
N–anterior nasal spine (N–ANS)	The distance measured from N to ANS
ANS–Me	The distance measured from ANS to Me
S–Go	The linear distance between sella and Go
Sella–posterior nasal spine (S–PNS)	The distance measured from sella to PNS
PNS–Go	The distance measured from posterior nasal point to Go point

Note: Angular variables are measured in degrees, and linear variables are measured in mm (except Jarabak's ratio).

Statistical analysis was done by using IBM SPSS (Statistical Package for Social Sciences, Chicago, United States) version 25.0. Paired sample *t* -tests were used to determine significant changes produced by RFA and FFA individually. Furthermore, nonparametric Mann–Whitney *U* -test was used to evaluate the intergroup differences at each time point interpreted at the 5% ( *p* < 0.05) significance level.

## Results

Thirty patients, 16 males (53.3%) and 14 females (46.6%), were recruited in two equal groups, one was treated with twin block (RFA) and the other with AdvanSync2 (FFA). Both the appliance groups showed positive and somewhat similar changes in linear and angular variables when comparison between T _1_ and T _2_ was made individually using paired *t* -test. Table 2 shows changes exhibited by the two groups which include significant decrease in ANB angle (RFA, *p* = 0.000; FFA, *p* = 0.001), a significant increase in the value of SNB angle ( *p* = 0.006 and 0.000 for RFA and FFA, respectively), significant increase in mandible length indicated by MCL and gonion–menton (Go–Me) values; a significant increase in nasion–Me (N–Me) pointed an increase in mandibular height which ultimately improved facial profile.

**Table 2:** Changes produced in RFA and FFA between pretreatment (T _1_ ) and posttreatment (T _2_ )

Variables	Parameters	RFA			FFA		
		T _1_ ( *n* = 15)	T _2_ ( *n* = 15)	*p* -Value	T _1_ ( *n* = 15)	T _2_ ( *n* = 15)	*p* -Value
		Mean ± SD	Mean ± SD		Mean ± SD	Mean ± SD	
Angular (degree)	SNA	81.20 ± 1.897	80.27 ± 2.915	0.084	80.13 ± 3.378	79.50 ± 2.667	0.120
	SNB	74.60 ± 2.586	76.20 ± 2.145	0.006 b	74.47 ± 1.959	75.67 ± 1.839	0.000 c
	ANB	6.93 ± 1.981	4.33 ± 1.543	0.000 c	5.53 ± 1.995	4.00 ± 1.690	0.001 b
	SN–MP	29.40 ± 3.019	30.13 ± 2.532	0.246	29.33 ± 4.287	29.93 ± 3.731	0.279
	SN–PP	7.73 ± 3.731	9.13 ± 3.137	0.022 a	9.73 ± 1.100	10.93 ± 2.374	0.051
	SN–OP	19.87 ± 4.454	18.53 ± 5.027	0.203	19.93 ± 3.788	19.60 ± 3.738	0.628
	Saddle	124.00 ± 6.708	124.60 ± 4.273	0.619	125.53 ± 8.114	124.93 ± 8.172	0.412
	Ar	143.67 ± 6.332	141.80 ± 8.172	0.341	140.20 ± 11.194	140.20 ± 11.645	1.000
	Gonial angle	124.07 ± 6.041	121.87 ± 6.289	0.076	122.67 ± 6.298	123.47 ± 6.643	0.420
	Y-axis	66.27 ± 4.026	67.07 ± 3.173	0.276	67.87 ± 3.114	68.07 ± 2.086	0.607
	UI–SN	113.73 ± 4.667	113.80 ± 4.427	0.937	111.67 ± 6.241	110.33 ± 4.655	0.232
	UI–PP	121.07 ± 3.348	121.33 ± 3.885	0.735	119.93 ± 7.421	119.00 ± 7.010	0.380
	IMPA	102.07 ± 7.526	102.07 ± 2.840	1.000	99.87 ± 4.897	102.00 ± 4.957	0.006 b
	IIA	115.13 ± 5.951	114.00 ± 5.264	0.171	118.47 ± 10.169	116.80 ± 7.912	0.180
Linear (mm except Jarabak's ratio)	SN	59.53 ± 2.503	60.00 ± 2.726	0.014 a	59.93 ± 1.580	60.13 ± 1.642	0.189
	MCL	58.20 ± 4.724	59.93 ± 4.788	0.000 c	56.60 ± 5.742	58.93 ± 5.298	0.001 b
	S–Ar	30.40 ± 3.501	30.73 ± 3.305	0.571	28.60 ± 1.765	29.53 ± 1.807	0.079
	Ar–Go	36.00 ± 3.381	38.93 ± 3.150	0.000 c	37.73 ± 3.390	38.80 ± 2.624	0.211
	Go–Me	55.87 ± 5.951	57.73 ± 5.837	0.000 c	54.87 ± 5.896	57.13 ± 6.523	0.002 b
	N–Me	95.40 ± 4.222	98.53 ± 4.340	0.000 c	96.27 ± 4.317	97.93 ± 3.788	0.001 b
	N–ANS	44.13 ± 2.386	45.00 ± 2.478	0.155	44.00 ± 2.138	44.67 ± 2.093	0.146
	ANS–Me	53.93 ± 5.035	54.80 ± 4.754	0.078	52.67 ± 4.850	52.67 ± 3.478	1.000
	S–Go	63.40 ± 3.397	65.33 ± 3.374	0.002 b	62.47 ± 3.420	62.80 ± 3.385	0.654
	S–PNS	40.07 ± 2.915	40.93 ± 3.283	0.072	38.93 ± 7.166	40.00 ± 6.176	0.037 a
	PNS–Go	37.20 ± 3.550	38.40 ± 5.539	0.343	37.47 ± 3.137	38.27 ± 3.390	0.118
	Jarabak's ratio	65.33 ± 3.177	65.87 ± 2.295	0.502	64.60 ± 3.961	63.67 ± 3.457	0.270

Abbreviations: A, subspinale; ANS, anterior nasal spine; Ar, articulare; B, supramentale; FFA, fixed functional appliances (AdvanSync2); Gn, gnathion; Go, gonion; I, incisor; IIA, inter incisor angle; IMPA, incisor mandibular plane angle MCL, mandibular corpus length; Me, menton; MP, mandibular plane; N, nasion; OP, occlusal plane; PP, palatal plane; PNS, posterior nasal spine; RFA, removable functional appliances (twin block); S, sella; SD, standard deviation; SN, sella–nasion; SNA, SN point A; SNB, SN point B; UI, upper incisor.

Note: Paired sample *t* -test to detect changes in each group.

a *p* ≤ 0.05.

b *p* ≤ 0.01.

c *p* ≤ 0.001.

In contrast, differences were also noticed as significant increase in values of SN–palatal plane (PP; *p* = 0.022), SN length ( *p* = 0.014), articulare–Go (Ar–Go) length ( *p* = 0.000), and sella–Go (S–Go) length ( *p* = 0.002) that were observed only in case of RFA group, while incisor mandibular plane angle (IMPA; *p* = 0.006) and S–PNS ( *p* = 0.037) values increased significantly for FFA group

The comparison between the two RFA and FFA groups before treatment (T _1_ ) and after treatment (T _2_ ) is illustrated in Table 3 . Only single parameter was significantly different between the two groups at T _1_ , that is, S–PNS value was significantly greater ( *p* = 0.019) for RFA (40.07 mm ± 2.915) group than FFA (38.93 mm ± 7.166) group. *p* -Value for all the other variables was greater than 0.05 indicating no significant difference between the two treatment groups at T _1_ .

**Table 3:** Comparison of changes between RFA and FFA groups at pretreatment (T _1_ ) and posttreatment (T _2_ )

Variables	Parameters	T _1_			T _2_		
		RFA ( *n* = 15)	FFA ( *n* = 15)	*p* -Value	RFA ( *n* = 15)	FFA ( *n* = 15)	*p* -Value
		Mean ± SD	Mean ± SD		Mean ± SD	Mean ± SD	
Angular (degree)	SNA	81.20 ± 1.897	80.13 ± 3.378	0.567	80.27 ± 2.915	79.50 ± 2.667	0.436
	SNB	74.60 ± 2.586	74.47 ± 1.959	0.935	76.20 ± 2.145	75.67 ± 1.839	0.775
	ANB	6.93 ± 1.981	5.53 ± 1.995	0.161	4.33 ± 1.543	4.00 ± 1.690	0.412
	SN–MP	29.40 ± 3.019	29.33 ± 4.287	0.967	30.13 ± 2.532	29.93 ± 3.731	0.838
	SN–PP	7.73 ± 3.731	9.73 ± 1.100	0.174	9.13 ± 3.137	10.93 ± 2.374	0.148
	SN–OP	19.87 ± 4.454	19.93 ± 3.788	0.935	18.53 ± 5.027	19.60 ± 3.738	0.367
	Saddle	124.00 ± 6.708	125.53 ± 8.114	0.653	124.60 ± 4.273	124.93 ± 8.172	1.000
	Ar	143.67 ± 6.332	140.20 ± 11.194	0.775	141.80 ± 8.172	140.20 ± 11.645	0.653
	Gonial angle	124.07 ± 6.041	122.67 ± 6.298	0.512	121.87 ± 6.289	123.47 ± 6.643	0.486
	Y-axis	66.27 ± 4.026	67.87 ± 3.114	0.267	67.07 ± 3.173	68.07 ± 2.086	0.089
	UI–SN	113.73 ± 4.667	111.67 ± 6.241	0.285	113.80 ± 4.427	110.33 ± 4.655	0.050
	UI–PP	121.07 ± 3.348	119.93 ± 7.421	0.461	121.33 ± 3.885	119.00 ± 7.010	0.683
	IMPA	102.07 ± 7.526	99.87 ± 4.897	0.539	102.07 ± 2.840	102.00 ± 4.957	1.000
	IIA	115.13 ± 5.951	118.47 ± 10.169	0.595	114.00 ± 5.264	116.80 ± 7.912	0.325
Linear (mm)	SN	59.53 ± 2.503	59.93 ± 1.580	1.000	60.00 ± 2.726	60.13 ± 1.642	0.595
	MCL	58.20 ± 4.724	56.60 ± 5.742	0.305	59.93 ± 4.788	58.93 ± 5.298	0.567
	S–Ar	30.40 ± 3.501	28.60 ± 1.765	0.148	30.73 ± 3.305	29.53 ± 1.807	0.713
	Ar–Go	36.00 ± 3.381	37.73 ± 3.390	0.187	38.93 ± 3.150	38.80 ± 2.624	0.870
	Go–Me	55.87 ± 5.951	54.87 ± 5.896	0.967	57.73 ± 5.837	57.13 ± 6.523	1.000
	N–Me	95.40 ± 4.222	96.27 ± 4.317	0.412	98.53 ± 4.340	97.93 ± 3.788	0.775
	N–ANS	44.13 ± 2.386	44.00 ± 2.138	0.935	45.00 ± 2.478	44.67 ± 2.093	0.838
	ANS–Me	53.93 ± 5.035	52.67 ± 4.850	0.595	54.80 ± 4.754	52.67 ± 3.478	0.137
	S–Go	63.40 ± 3.397	62.47 ± 3.420	0.567	65.33 ± 3.374	62.80 ± 3.385	0.067
	S–PNS	40.07 ± 2.915	38.93 ± 7.166	0.019 a	40.93 ± 3.283	40.00 ± 6.176	0.010 a
	PNS–Go	37.20 ± 3.550	37.47 ± 3.137	1.000	38.40 ± 5.539	38.27 ± 3.390	0.775
	Jarabak's ratio	65.33 ± 3.177	64.60 ± 3.961	0.325	65.87 ± 2.295	63.67 ± 3.457	0.045 a

Abbreviations: A, subspinale; ANS, anterior nasal spine; Ar, articulare; B, supramentale; FFA, fixed functional appliances (AdvanSync2); Gn, gnathion; Go, gonion; I, incisor; IIA, inter incisor angle; IMPA, incisor mandibular plane angle MCL, mandibular corpus length; Me, menton; MP, mandibular plane; N, nasion; OP, occlusal plane; PP, palatal plane; PNS, posterior nasal spine; RFA, removable functional appliances (twin block); S, sella; SD, standard deviation; SN, sella–nasion; SNA, SN point A; SNB, SN point B; UI, upper incisor.

a *p* ≤ 0.05.

At T _2_ , two variables showed significant differences among RFA and FFA groups. S–PNS length significantly increased and Jarabak's ratio significantly decreased for FFA group ( *p* = 0.010 and 0.045, respectively) when compared with the values obtained from RFA group.

## Discussion

Functional appliances (removable or fixed) are used during growth spurts (active growth periods) with the purpose to bring about maximum amount of skeletal change. Thus, to achieve maximum therapeutic effects of both the appliances, treatment involved inclusion of growth spurts. 12 Nevertheless, the amount of skeletal or dental change cannot be quantified with ease, as it relies on various external and internal factors. 13

In this study, comparison of twin block appliance (RFA) with AdvanSync2 appliance (FFA) has been made. These modalities are used for correction of class-II malocclusion. The findings of current study revealed that both appliances can be used to correct this type of malocclusion effectively and efficiently. This conclusion appears obvious from the ANB angle values that significantly decreased for both the groups from skeletal class-II to class-I pattern in sagittal plane.

For the skeletal analysis in sagittal plane, maxillary and mandibular components are determined by SN point A (SNA) and SNB, respectively. ANB determines maxillomandibular relationship. First, mean of SNA for RFA (81.20 ± 1.897) and FFA (80.13 ± 3.378) indicated that maxilla was in normal relationship with the anterior cranial base at T _1_ (pretreatment). Moreover, SNB mean for RFA and FFA at T _1_ were found to be 74.60 ± 2.586 and 74.47 ± 1.959, respectively, indicating mandibular retrusion. Both the appliance groups showed positive and somewhat similar changes in linear and angular variables after functional jaw orthopedics treatment (T _2_ ). Significant changes observed in the study due to forced forward position of the mandible including decrease in ANB angle (RFA, *p* = 0.000; FFA, *p* = 0.001) mainly due to an increase in the value of SNB angle ( *p* = 0.006 and *p* = 0.000 for RFA and FFA, respectively). The results are consistent with previous studies for RFA group 14 15 but this significant change has not been reported for FFA group. 13 A significant increase in mandible length was observed in both groups indicated by MCL (RFA, *p* = 0.000; FFA, *p* = 0.001) and Go–Me values (RFA, *p* = 0.000; FFA, *p* = 0.002). This finding is in agreement with the studies that reported increase in mandibular length. 15 16 Conversely, some of the studies reported no increase in mandibular length but found significant increase in its height. 17 A significant increase in N–Me was also noticed (RFA, *p* = 0.000; FFA, *p* = 0.001) which indicated an increase in vertical growth in our study.

Functional appliances are known to produce distalizing effect on maxilla while promoting forward mandibular movement. 18 No significant restrictive maxillary effect was noticed for both the appliances in our study, although SNA angle decreased, so there might be some retrusive action on maxilla especially for FFA group. The evidence is controversial in this regard as some studies reported restriction of maxilla, 13 19 20 21 22 23 while the others showed no such effect. 24 25 The difference in the results could be due to variation in working methods for mandibular advancement as some investigators preferred incremental advancement (3–4 mm), while others advanced the mandible to its maximum limit (7 mm).

In contrast to similar findings for both the groups, some differences were also noticed which include significant increase in values of SN–PP ( *p* = 0.022), Ar–Go length ( *p* = 0.000), and S–Go length ( *p* = 0.002) that were observed only in case of RFA group. These findings are consistent with several studies that reported an increase in lower anterior and posterior facial heights after removable twin block therapy. 16 17 19 26 27 Acrylic bite blocks contouring should be taken into considerations when an increase in lower anterior facial height is desired, like in current study, we sequentially trimmed the bite blocks in low-angle (deep-bite) patients to increase the vertical dimensions. On the contrary, a “posterior bite-block effect” may be utilized that may inhibit vertical growth, if not trimmed. 19 Thus, the results observed in our study should be cautiously interpreted.

The inclinations of lower incisors significantly increased with AdvanSync2 indicated by IMPA ( *p* = 0.006), while they were insignificant for RFA group. As reported previously, the AdvanSync2, which is a molar-to-molar attachment produces sagittal, intrusive, and expansive force vectors with a combination of mandibular molar mesialization and mild lower incisor proclination, 13 is in accordance with our readings. The inclinations can be controlled using fixed appliances with labial root torque, cinching back the wire or using heavy stabilizing arch wires, and enhancing anchorage using miniscrews as suggested by previous studies. 28 29

When comparison was made between RFA and FFA groups, no significant differences were found in the sagittal plane. This may be because of their homogenous mechanism of action. S–PNS length significantly increased and Jarabak's ratio significantly decreased for FFA group ( *p* = 0.010 and 0.045, respectively) when compare with RFA group. These changes, which are in agreement with a recent study, 13 define clockwise rotation induced in the mandible that may motivate vertical growth.

AdvanSync2 is a type of FFA which does not require preorthodontic alignment prior to its fixation in oral cavity, so capitalization of growth can be easily achieved along with favorable shorter treatment duration in patients who have passed their peak height velocity growth spurt. Since it is a new treatment modality, little work has been done regarding this appliance, whereas a lot of literature is available on other FFA, for example, Herbst appliance of which AdvanSync2 is a modified version that shows dentoskeletal, as well as soft tissue changes, like Herbst appliance, thus a similar stable treatment result can be expected. 30 Results were found to be similar in terms of patient compliance and duration of treatment when the Frankel-2 appliance compared with twin block appliance. 31 The changes in terms of maxillary and mandibular movements were observed to be better with the Herbst appliance as compare with that of twin block. 32 Previous studies has supported that Herbst appliances in combination with edge wise brackets contributes more toward perseverance to achieve the required skeletal changes. 33 AdvanSync2 appliance, in contrast to MARA, is known to show more of headgear effects. However dentoalveolar changes with both the appliances were found to be similar. 34

Frequent band dislodgements were faced for which recementation of bulky bands had to be employed. A major limitation of our research was a lack of untreated control group, thus resulting changes cannot only be attributed to treatment but also to residual growth. Further longitudinal researches are required in this perspective.

## Conclusion

Therapies with both the appliances, twin block (RFA) and AdvanSync2 (FFA), were found to be effective for treating class-II malocclusions, including significant forward mandibular advancement with subsequent improvement of skeletal discrepancy. Both the appliances produced similar effects in the sagittal plane but some maxillary restriction was observed for AdvanSync2 appliance. The statistically significant differences recorded between the treatment groups were lower incisors proclinations increased with FFA along with clockwise rotation of the mandible that improved facial profile of patients. Verticals were maintained with RFA which can be increased with sequential trimming when required (deep bite correction). Hence, twin block can be used to inhibit vertical development, while AdvanSync2 can be used to produce significant mandibular changes in individuals with postpubertal growth past peak height velocity.
